# Methyl 2-((2*Z*,5*Z*)-2-{(*E*)-2-[1-(4-hy­droxy­phen­yl)ethyl­idene]hydrazin-1-yl­idene}-4-oxo-3-phenyl-1,3-thia­zolidin-5-yl­idene)acetate

**DOI:** 10.1107/S1600536813025270

**Published:** 2013-09-18

**Authors:** Shaaban K. Mohamed, Joel T. Mague, Mehmet Akkurt, Alaa A. Hassan, Mustafa R. Albayati

**Affiliations:** a Chemistry and Environmental Division, Manchester Metropolitan University, Manchester, M1 5GD, England; b Chemistry Department, Faculty of Science, Minia University, 61519 El-Minia, Egypt; c Department of Chemistry, Tulane University, New Orleans, LA 70118, USA; d Department of Physics, Faculty of Sciences, Erciyes University, 38039 Kayseri, Turkey; e Kirkuk University, College of Science, Department of Chemistry, Kirkuk, Iraq

## Abstract

In the title compound, C_20_H_17_N_3_O_4_S, all non-H atoms, except those of the phenyl ring, are approximately coplanar [maximum deviation = 0.2214 (1) Å], and the dihedral angle between their best plane and the benzene ring is 53.13 (1)°. A short intra­molecular O⋯S contact of 2.838 (1) Å is formed between the ester carbonyl O atom and the S atom of the thia­zolidine ring. In the crystal, mol­ecules associated *via* O—H⋯O, C—H⋯O and C—H⋯S hydrogen bonds form layers parallel to (010), with only C—H⋯O-type short contacts between the mol­ecules in adjacent layers.

## Related literature

For the biological activity of 4-thia­zolidinones, see: Dayam *et al.* (2006 ▶); Srivastava *et al.* (2005 ▶), Look *et al.* (1996 ▶), Barreca *et al.* (2001 ▶); Diurno *et al.* (1992 ▶).
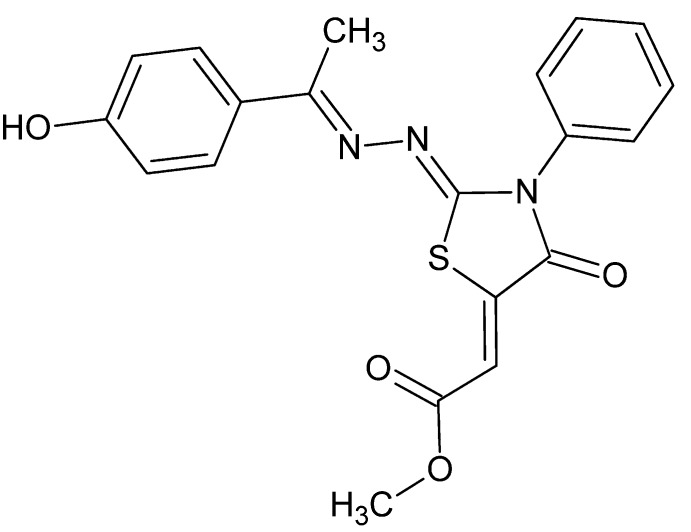


## Experimental

### 

#### Crystal data

C_20_H_17_N_3_O_4_S
*M* *_r_* = 395.42
Monoclinic, 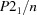

*a* = 9.5049 (9) Å
*b* = 20.656 (2) Å
*c* = 10.1364 (10) Å
β = 107.637 (1)°
*V* = 1896.6 (3) Å^3^
*Z* = 4
Mo *K*α radiation
μ = 0.20 mm^−1^
*T* = 150 K
0.19 × 0.11 × 0.05 mm

#### Data collection

Bruker SMART APEX CCD diffractometer
Absorption correction: multi-scan (*SADABS*; Bruker, 2013 ▶) *T*_min_ = 0.82, *T*_max_ = 0.99
16907 measured reflections
4582 independent reflections
3740 reflections with i > 2σ(i)
*R*_int_ = 0.039

#### Refinement

*R*[*F* ^2^ > 2σ(*F* ^2^)] = 0.044
*wR*(*F* ^2^) = 0.118
*S* = 1.06
4582 reflections
259 parameters
H atoms treated by a mixture of independent and constrained refinement
Δρ_max_ = 0.34 e Å^−3^
Δρ_min_ = −0.44 e Å^−3^

### 

Data collection: *APEX2* (Bruker, 2013 ▶); cell refinement: *SAINT* (Bruker, 2013 ▶); data reduction: *SAINT*; program(s) used to solve structure: *SHELXT* (Sheldrick, 2008 ▶); program(s) used to refine structure: *SHELXL2013* (Sheldrick, 2008 ▶); molecular graphics: *ORTEP-3 for Windows* (Farrugia, 2012 ▶); software used to prepare material for publication: *WinGX* (Farrugia, 2012 ▶) and *PLATON* (Spek, 2009 ▶).

## Supplementary Material

Crystal structure: contains datablock(s) global, I. DOI: 10.1107/S1600536813025270/gk2589sup1.cif

Structure factors: contains datablock(s) I. DOI: 10.1107/S1600536813025270/gk2589Isup2.hkl

Supplementary material file. DOI: 10.1107/S1600536813025270/gk2589Isup3.cml

Additional supplementary materials:  crystallographic information; 3D view; checkCIF report

## Figures and Tables

**Table 1:** Hydrogen-bond geometry (Å, °)

*D*—H⋯*A*	*D*—H	H⋯*A*	*D*⋯*A*	*D*—H⋯*A*
O4—H4*O*⋯O1^i^	0.84 (2)	1.96 (2)	2.7901 (16)	174.8 (19)
C8—H8⋯S1^ii^	0.95	2.82	3.7272 (17)	160
C10—H10⋯O4^iii^	0.95	2.52	3.452 (2)	167
C19—H19⋯O1^i^	0.95	2.47	3.200 (2)	133

Symmetry codes: (i) 
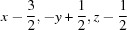
; (ii) 
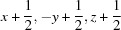
; (iii) 
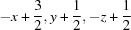
.

**Fig. 1.**
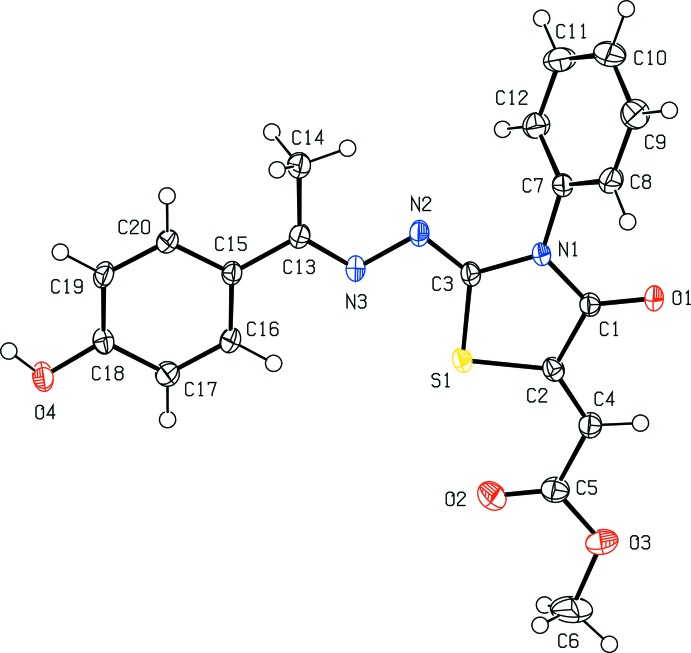
Perspective view of the title molecule with 50% probability displacement ellipsoids.

**Fig. 2.**
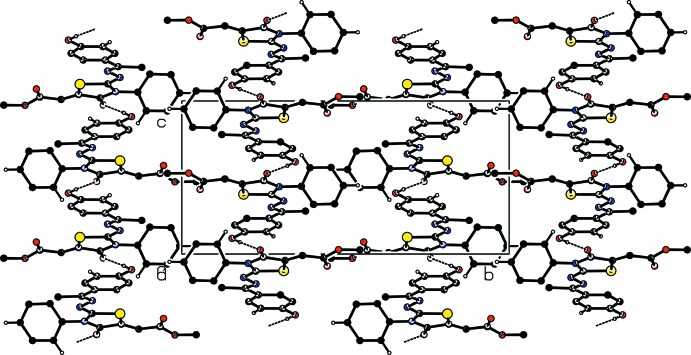
View of the crystal packing down the a-axis showing the layer structure and O-H···O hydrogen bonding interactions as dotted lines.

## supplementary crystallographic information

### 1. Comment

Thiazolidinone scaffold compounds have received much attention from organic and medicinal chemists due to their therapeutic diversity coupled with their commercial viability. Recently, 4-thiazolidinones have exhibited many interesting bio-activity profiles such as anti-cancer (Dayam *et al.*, 2006) and anti-mycobacterial agents (Srivastava *et al.*, 2005), COX-1 inhibitors (Look *et al.* 1996), non-nucleoside inhibitors of HIV-RT (Barreca *et al.*, 2001) and anti-histaminic agents (Diurno *et al*., 1992). In view of these properties the title compound has been synthesized among a series of other 4-thiazolidinones to investigate the relationship between their crystal structures and their antibacterial activity.

In the title compound (Fig. 1), all non-H atoms, except the phenyl group (C7–C12), are approximately coplanar, with the maximum deviations of -0.2214 (1) Å for C6, -0.2097 (1) Å for C14, 0.1651 (1) Å for O2 and -0.1009 (1) Å for O3, and the benzene ring (C7–C12) makes a dihedral angle of 53.13 (1)° with this plane. Molecular conformation is stabilized by a short intramolecular O···S contact of 2.838 (1) Å.

The title compound crystallizes in a layer structure with the layers parallel to the (010) plane (Fig. 2). Molecules within the layers are associated *via* O—H···O, C—H···O and C—H···S hydrogen bonding (Table 1, Fig. 2). One of the C—H···O contacts (C10—H10···O4) in Table 1 is between the layers. The interlayer regions are occupied by the *N*-phenyl and ester groups between which there are no significant interactions.

### 2. Experimental

A mixture of 283 mg (1 mmol) (2*Z*)-2-[1-(4-methylphenyl)ethylidene]-*N*-phenylhydrazinecarbothioamide and 142 mg (1 mmol) dimethyl but-2-ynedioate in 50 ml of ethanol was refluxed and monitored by TLC until completion of the reaction. The excess solvent was evaporated under *vacuum* and the solid obtained was recrystallized from ethanol to afford clear yellow plates (*M*.p. 541–543 K) of X-ray quality.

### 3. Refinement

The hydroxyl H atom was found from a difference Fourier map [O4—H4O = 0.84 (2) Å] and refined freely. H atoms bonded to C were placed in geometrically idealized positions and constrained to ride on their parent atoms C—H = 0.95 Å (aromatic H) and 0.98 Å (methyl H), with *U*_iso_(H) = 1.5 *U*_iso_(C) for methyl H atoms and *U*_iso_(H) = 1.2 *U*_iso_(C) for other H atoms.

### Crystal data

**Table:**

C_20_H_17_N_3_O_4_S	*F*(000) = 824
*M**_r_* = 395.42	*D*_x_ = 1.385 Mg m^−^^3^
Monoclinic, *P*2_1_/*n*	Mo *K*α radiation, λ = 0.71073 Å
Hall symbol: -P 2yn	Cell parameters from 8755 reflections
*a* = 9.5049 (9) Å	θ = 2.3–28.6°
*b* = 20.656 (2) Å	µ = 0.20 mm^−^^1^
*c* = 10.1364 (10) Å	*T* = 150 K
β = 107.637 (1)°	Plate, clear yellow
*V* = 1896.6 (3) Å^3^	0.19 × 0.11 × 0.05 mm
*Z* = 4	

### Data collection

**Table:**

Bruker SMART APEX CCD diffractometer	4582 independent reflections
Radiation source: fine-focus sealed tube	3740 reflections with i > 2σ(i)
Graphite monochromator	*R*_int_ = 0.039
Detector resolution: 8.3660 pixels mm^-1^	θ_max_ = 28.7°, θ_min_ = 2.0°
φ and ω scans	*h* = −12→12
Absorption correction: multi-scan (*SADABS*; Bruker, 2013)	*k* = −27→27
*T*_min_ = 0.82, *T*_max_ = 0.99	*l* = −13→13
16907 measured reflections	

### Refinement

**Table:**

Refinement on *F*^2^	Primary atom site location: structure-invariant direct methods
Least-squares matrix: full	Secondary atom site location: difference Fourier map
*R*[*F*^2^ > 2σ(*F*^2^)] = 0.044	H atoms treated by a mixture of independent and constrained refinement
*wR*(*F*^2^) = 0.118	W = 1/[Σ^2^(*F*O^2^) + (0.0643*P*)^2^ + 0.3949*P*] WHERE *P* = (*F*O^2^ + 2*F*C^2^)/3
*S* = 1.06	(Δ/σ)_max_ = 0.001
4582 reflections	Δρ_max_ = 0.34 e Å^−^^3^
259 parameters	Δρ_min_ = −0.44 e Å^−^^3^
0 restraints	

### Special details

**Table:**

Experimental. The diffraction data were collected in three sets of 606 frames (0.3° width in ω) at φ = 0, 120 and 240°. A scan time of 40 sec/frame was used.
Geometry. Bond distances, angles *etc*. have been calculated using the rounded fractional coordinates. All su's are estimated from the variances of the (full) variance-covariance matrix. The cell e.s.d.'s are taken into account in the estimation of distances, angles and torsion angles
Refinement. Refinement on *F*^2^ for ALL reflections except those flagged by the user for potential systematic errors. Weighted *R*-factors *wR* and all goodnesses of fit *S* are based on *F*^2^, conventional *R*-factors *R* are based on *F*, with *F* set to zero for negative *F*^2^. The observed criterion of *F*^2^ > σ(*F*^2^) is used only for calculating -*R*-factor-obs *etc*. and is not relevant to the choice of reflections for refinement. *R*-factors based on *F*^2^ are statistically about twice as large as those based on *F*, and *R*-factors based on ALL data will be even larger.

### Fractional atomic coordinates and isotropic or equivalent isotropic displacement parameters (Å^2^)

**Table:**

	*x*	*y*	*z*	*U*_iso_*/*U*_eq_	
S1	1.01222 (4)	0.18869 (2)	0.39033 (4)	0.0203 (1)	
O1	1.40358 (11)	0.26023 (5)	0.52637 (11)	0.0240 (3)	
O2	1.09079 (13)	0.05575 (6)	0.42140 (13)	0.0347 (4)	
O3	1.32263 (14)	0.02111 (6)	0.52834 (14)	0.0378 (4)	
O4	0.14035 (12)	0.15348 (6)	0.10230 (13)	0.0285 (3)	
N1	1.16412 (13)	0.29748 (6)	0.43935 (12)	0.0189 (3)	
N2	0.90771 (13)	0.31123 (6)	0.34670 (13)	0.0222 (4)	
N3	0.77907 (13)	0.27335 (6)	0.30473 (13)	0.0219 (4)	
C1	1.27118 (16)	0.25046 (7)	0.47990 (15)	0.0190 (4)	
C2	1.20296 (16)	0.18459 (7)	0.45802 (14)	0.0193 (4)	
C3	1.01955 (15)	0.27395 (7)	0.38850 (14)	0.0190 (4)	
C4	1.28646 (17)	0.13149 (7)	0.49043 (16)	0.0229 (4)	
C5	1.22088 (18)	0.06672 (8)	0.47422 (16)	0.0260 (5)	
C6	1.2661 (3)	−0.04389 (9)	0.5273 (3)	0.0553 (8)	
C7	1.20015 (15)	0.36539 (7)	0.44261 (16)	0.0206 (4)	
C8	1.29110 (17)	0.39165 (8)	0.56415 (17)	0.0275 (5)	
C9	1.3342 (2)	0.45594 (9)	0.5655 (2)	0.0368 (5)	
C10	1.2839 (2)	0.49351 (8)	0.4477 (2)	0.0384 (6)	
C11	1.1899 (2)	0.46687 (8)	0.32791 (19)	0.0347 (5)	
C12	1.14814 (18)	0.40248 (8)	0.32417 (16)	0.0272 (5)	
C13	0.65717 (16)	0.30559 (7)	0.27572 (15)	0.0196 (4)	
C14	0.64599 (17)	0.37782 (8)	0.28058 (17)	0.0260 (5)	
C15	0.52047 (15)	0.26621 (7)	0.23315 (15)	0.0187 (4)	
C16	0.52602 (16)	0.19872 (7)	0.23265 (17)	0.0241 (4)	
C17	0.39928 (17)	0.16202 (8)	0.18891 (18)	0.0267 (5)	
C18	0.26171 (16)	0.19204 (7)	0.14442 (15)	0.0207 (4)	
C19	0.25339 (16)	0.25898 (7)	0.14520 (16)	0.0227 (4)	
C20	0.38175 (16)	0.29540 (7)	0.18934 (16)	0.0221 (4)	
H4	1.39070	0.13580	0.52480	0.0270*	
H4O	0.066 (2)	0.1774 (11)	0.077 (2)	0.046 (6)*	
H6A	1.19470	−0.04490	0.57960	0.0830*	
H6B	1.34780	−0.07350	0.56990	0.0830*	
H6C	1.21740	−0.05730	0.43150	0.0830*	
H8	1.32360	0.36600	0.64560	0.0330*	
H9	1.39860	0.47420	0.64770	0.0440*	
H10	1.31370	0.53750	0.44900	0.0460*	
H11	1.15380	0.49300	0.24760	0.0420*	
H12	1.08480	0.38410	0.24160	0.0330*	
H14A	0.74530	0.39660	0.31100	0.0390*	
H14B	0.59250	0.38990	0.34570	0.0390*	
H14C	0.59280	0.39420	0.18820	0.0390*	
H16	0.61910	0.17760	0.26310	0.0290*	
H17	0.40570	0.11610	0.18910	0.0320*	
H19	0.16000	0.27990	0.11560	0.0270*	
H20	0.37500	0.34130	0.18970	0.0260*	

### Atomic displacement parameters (Å^2^)

**Table:**

	*U*^11^	*U*^22^	*U*^33^	*U*^12^	*U*^13^	*U*^23^
S1	0.0149 (2)	0.0209 (2)	0.0233 (2)	−0.0034 (1)	0.0030 (1)	−0.0019 (1)
O1	0.0134 (5)	0.0242 (6)	0.0308 (6)	−0.0009 (4)	0.0011 (4)	0.0019 (4)
O2	0.0316 (7)	0.0275 (6)	0.0427 (7)	−0.0071 (5)	0.0078 (5)	−0.0030 (5)
O3	0.0387 (7)	0.0198 (6)	0.0525 (8)	0.0051 (5)	0.0104 (6)	0.0035 (5)
O4	0.0159 (5)	0.0239 (6)	0.0441 (7)	−0.0016 (5)	0.0067 (5)	−0.0074 (5)
N1	0.0131 (6)	0.0191 (6)	0.0222 (6)	−0.0025 (5)	0.0017 (5)	−0.0002 (5)
N2	0.0141 (6)	0.0247 (7)	0.0252 (7)	−0.0007 (5)	0.0022 (5)	−0.0009 (5)
N3	0.0137 (6)	0.0252 (7)	0.0250 (7)	−0.0005 (5)	0.0030 (5)	0.0008 (5)
C1	0.0158 (7)	0.0215 (7)	0.0187 (7)	−0.0012 (5)	0.0036 (5)	0.0005 (5)
C2	0.0180 (7)	0.0220 (7)	0.0172 (7)	−0.0034 (6)	0.0043 (5)	−0.0011 (5)
C3	0.0147 (7)	0.0220 (7)	0.0186 (7)	−0.0026 (5)	0.0025 (5)	−0.0012 (5)
C4	0.0193 (7)	0.0236 (8)	0.0242 (7)	0.0000 (6)	0.0044 (6)	−0.0009 (6)
C5	0.0294 (8)	0.0222 (8)	0.0273 (8)	0.0005 (6)	0.0101 (7)	−0.0014 (6)
C6	0.0658 (15)	0.0195 (9)	0.0817 (17)	0.0013 (9)	0.0238 (13)	0.0051 (10)
C7	0.0153 (6)	0.0181 (7)	0.0273 (8)	−0.0001 (5)	0.0050 (6)	−0.0002 (6)
C8	0.0230 (8)	0.0242 (8)	0.0296 (8)	0.0001 (6)	−0.0005 (6)	0.0014 (6)
C9	0.0334 (9)	0.0251 (9)	0.0423 (10)	−0.0044 (7)	−0.0031 (8)	−0.0061 (7)
C10	0.0401 (10)	0.0182 (8)	0.0524 (12)	−0.0038 (7)	0.0071 (9)	0.0001 (7)
C11	0.0408 (10)	0.0246 (8)	0.0354 (9)	0.0018 (7)	0.0066 (8)	0.0074 (7)
C12	0.0283 (8)	0.0242 (8)	0.0251 (8)	−0.0008 (6)	0.0023 (6)	−0.0004 (6)
C13	0.0157 (7)	0.0239 (8)	0.0185 (7)	0.0017 (6)	0.0041 (5)	0.0016 (5)
C14	0.0198 (7)	0.0224 (8)	0.0344 (9)	0.0006 (6)	0.0060 (6)	−0.0006 (6)
C15	0.0143 (7)	0.0228 (7)	0.0190 (7)	0.0009 (5)	0.0051 (5)	0.0017 (5)
C16	0.0151 (7)	0.0232 (8)	0.0334 (8)	0.0042 (6)	0.0065 (6)	0.0029 (6)
C17	0.0206 (8)	0.0191 (7)	0.0409 (9)	0.0027 (6)	0.0099 (7)	−0.0003 (6)
C18	0.0162 (7)	0.0234 (8)	0.0230 (7)	−0.0013 (6)	0.0069 (6)	−0.0028 (6)
C19	0.0147 (7)	0.0249 (8)	0.0270 (8)	0.0044 (6)	0.0041 (6)	0.0003 (6)
C20	0.0171 (7)	0.0195 (7)	0.0289 (8)	0.0032 (6)	0.0059 (6)	0.0015 (6)

### Geometric parameters (Å, º)

**Table:**

S1—C2	1.7352 (16)	C13—C15	1.482 (2)
S1—C3	1.7628 (15)	C15—C20	1.394 (2)
O1—C1	1.2195 (19)	C15—C16	1.395 (2)
O2—C5	1.211 (2)	C16—C17	1.378 (2)
O3—C5	1.343 (2)	C17—C18	1.393 (2)
O3—C6	1.445 (2)	C18—C19	1.385 (2)
O4—C18	1.360 (2)	C19—C20	1.387 (2)
O4—H4O	0.84 (2)	C4—H4	0.9500
N1—C1	1.376 (2)	C6—H6A	0.9800
N1—C7	1.4420 (19)	C6—H6B	0.9800
N1—C3	1.4003 (19)	C6—H6C	0.9800
N2—N3	1.4046 (18)	C8—H8	0.9500
N2—C3	1.2766 (19)	C9—H9	0.9500
N3—C13	1.291 (2)	C10—H10	0.9500
C1—C2	1.495 (2)	C11—H11	0.9500
C2—C4	1.335 (2)	C12—H12	0.9500
C4—C5	1.464 (2)	C14—H14A	0.9800
C7—C12	1.383 (2)	C14—H14B	0.9800
C7—C8	1.384 (2)	C14—H14C	0.9800
C8—C9	1.389 (3)	C16—H16	0.9500
C9—C10	1.382 (3)	C17—H17	0.9500
C10—C11	1.385 (3)	C19—H19	0.9500
C11—C12	1.385 (2)	C20—H20	0.9500
C13—C14	1.498 (2)		
			
C2—S1—C3	90.75 (7)	O4—C18—C17	117.69 (13)
C5—O3—C6	115.39 (17)	O4—C18—C19	122.83 (14)
C18—O4—H4O	107.9 (15)	C17—C18—C19	119.48 (14)
C1—N1—C3	114.79 (12)	C18—C19—C20	119.81 (14)
C3—N1—C7	123.08 (12)	C15—C20—C19	121.51 (13)
C1—N1—C7	122.05 (13)	C2—C4—H4	119.00
N3—N2—C3	108.99 (12)	C5—C4—H4	119.00
N2—N3—C13	114.87 (12)	O3—C6—H6A	109.00
O1—C1—C2	123.95 (14)	O3—C6—H6B	109.00
N1—C1—C2	110.48 (13)	O3—C6—H6C	109.00
O1—C1—N1	125.57 (14)	H6A—C6—H6B	110.00
S1—C2—C4	127.54 (12)	H6A—C6—H6C	109.00
C1—C2—C4	120.83 (14)	H6B—C6—H6C	109.00
S1—C2—C1	111.63 (11)	C7—C8—H8	120.00
S1—C3—N2	125.07 (12)	C9—C8—H8	120.00
N1—C3—N2	122.57 (13)	C8—C9—H9	120.00
S1—C3—N1	112.36 (10)	C10—C9—H9	120.00
C2—C4—C5	121.46 (15)	C9—C10—H10	120.00
O2—C5—O3	124.20 (16)	C11—C10—H10	120.00
O2—C5—C4	124.31 (15)	C10—C11—H11	120.00
O3—C5—C4	111.46 (14)	C12—C11—H11	120.00
N1—C7—C12	119.94 (14)	C7—C12—H12	121.00
C8—C7—C12	121.22 (14)	C11—C12—H12	121.00
N1—C7—C8	118.80 (13)	C13—C14—H14A	109.00
C7—C8—C9	119.12 (15)	C13—C14—H14B	109.00
C8—C9—C10	120.31 (17)	C13—C14—H14C	109.00
C9—C10—C11	119.78 (16)	H14A—C14—H14B	110.00
C10—C11—C12	120.59 (16)	H14A—C14—H14C	109.00
C7—C12—C11	118.95 (15)	H14B—C14—H14C	109.00
N3—C13—C15	115.50 (13)	C15—C16—H16	119.00
C14—C13—C15	119.45 (13)	C17—C16—H16	119.00
N3—C13—C14	125.05 (14)	C16—C17—H17	120.00
C13—C15—C20	121.09 (13)	C18—C17—H17	120.00
C16—C15—C20	117.65 (14)	C18—C19—H19	120.00
C13—C15—C16	121.25 (14)	C20—C19—H19	120.00
C15—C16—C17	121.37 (15)	C15—C20—H20	119.00
C16—C17—C18	120.18 (15)	C19—C20—H20	119.00
			
C3—S1—C2—C1	0.49 (11)	S1—C2—C4—C5	−1.6 (2)
C3—S1—C2—C4	179.93 (14)	C1—C2—C4—C5	177.79 (14)
C2—S1—C3—N1	−0.41 (11)	C2—C4—C5—O2	6.1 (3)
C2—S1—C3—N2	179.83 (13)	C2—C4—C5—O3	−172.43 (14)
C6—O3—C5—O2	−3.4 (3)	N1—C7—C8—C9	175.77 (15)
C6—O3—C5—C4	175.15 (17)	C12—C7—C8—C9	−2.0 (3)
C3—N1—C1—O1	179.63 (14)	N1—C7—C12—C11	−176.95 (15)
C3—N1—C1—C2	0.14 (17)	C8—C7—C12—C11	0.8 (3)
C7—N1—C1—O1	2.8 (2)	C7—C8—C9—C10	1.5 (3)
C7—N1—C1—C2	−176.72 (12)	C8—C9—C10—C11	0.1 (3)
C1—N1—C3—S1	0.22 (15)	C9—C10—C11—C12	−1.3 (3)
C1—N1—C3—N2	179.98 (14)	C10—C11—C12—C7	0.9 (3)
C7—N1—C3—S1	177.05 (11)	N3—C13—C15—C16	3.4 (2)
C7—N1—C3—N2	−3.2 (2)	N3—C13—C15—C20	−175.24 (14)
C1—N1—C7—C8	−52.6 (2)	C14—C13—C15—C16	−177.37 (14)
C1—N1—C7—C12	125.14 (16)	C14—C13—C15—C20	4.0 (2)
C3—N1—C7—C8	130.78 (15)	C13—C15—C16—C17	−177.90 (15)
C3—N1—C7—C12	−51.5 (2)	C20—C15—C16—C17	0.8 (2)
C3—N2—N3—C13	172.11 (13)	C13—C15—C20—C19	177.96 (14)
N3—N2—C3—S1	0.83 (17)	C16—C15—C20—C19	−0.7 (2)
N3—N2—C3—N1	−178.90 (12)	C15—C16—C17—C18	−0.3 (3)
N2—N3—C13—C14	1.6 (2)	C16—C17—C18—O4	179.96 (15)
N2—N3—C13—C15	−179.18 (12)	C16—C17—C18—C19	−0.3 (2)
O1—C1—C2—S1	−179.96 (13)	O4—C18—C19—C20	−179.92 (14)
O1—C1—C2—C4	0.6 (2)	C17—C18—C19—C20	0.4 (2)
N1—C1—C2—S1	−0.45 (15)	C18—C19—C20—C15	0.1 (2)
N1—C1—C2—C4	−179.95 (15)		

### Hydrogen-bond geometry (Å, º)

**Table:**

*D*—H···*A*	*D*—H	H···*A*	*D*···*A*	*D*—H···*A*
O4—H4*O*···O1^i^	0.84 (2)	1.96 (2)	2.7901 (16)	174.8 (19)
C8—H8···S1^ii^	0.95	2.82	3.7272 (17)	160
C10—H10···O4^iii^	0.95	2.52	3.452 (2)	167
C14—H14*A*···N2	0.98	2.30	2.742 (2)	106
C19—H19···O1^i^	0.95	2.47	3.200 (2)	133

Symmetry codes: (i) *x*−3/2, −*y*+1/2, *z*−1/2; (ii) *x*+1/2, −*y*+1/2, *z*+1/2; (iii) −*x*+3/2, *y*+1/2, −*z*+1/2.
